# Cortical vein involvement and its influence in a cohort of adolescents with cerebral venous thrombosis

**DOI:** 10.1186/s12959-023-00521-3

**Published:** 2023-07-24

**Authors:** Lu Liu, Chenxia Zhou, Huimin Jiang, Huimin Wei, Yifan Zhou, Yan Wu, Kaiyuan Zhang, Chuanhui Li, Jiangang Duan, Ran Meng, Chen Zhou, Xunming Ji

**Affiliations:** 1 Department of Neurology, Xuanwu Hospital, Capital Medical University, Beijing, China; 2 Beijing Institute of Brain Disorders, Capital Medical University, Beijing, 100069 China; 3 Beijing Advanced Innovation Center for Big Data-Based Precision Medicine, School of Biological Science and Medical Engineering, Beihang University, Beijing, 100191 China; 4 Beijing Institute of Geriatrics, Xuanwu Hospital, Capital Medical University, Beijing, China; 5 Department of Radiology & Nuclear Medicine, Xuanwu Hospital, Capital Medical University, Beijing, China; 6 Department of Emergency, Xuanwu Hospital Capital Medical University, Beijing, China; 7 Department of Neurosurgery, Xuanwu Hospital, Capital Medical University, Beijing, China

**Keywords:** Cerebral venous thrombosis, Adolescent, Cortical vein, Edema, Deterioration

## Abstract

**Background and purpose:**

Cortical vein thrombosis (CVT) is a rare form of cerebral venous sinus thrombosis (CVST) in adolescent patients that has received little attention. We aimed to analyze the clinical and radiological features of adolescents with CVST and investigate the effects of CVT involvement.

**Methods:**

Patients aged ≥ 10 to ≤ 18 years and diagnosed with CVST were identified at Xuanwu Hospital, Capital Medical University between January 2015 and August 2022 and divided into two groups according to the presence or absence of cortical vein involvement. Additionally, the patients were also categorized based on their sex. Clinical features, radiological characteristics, and 12-month follow-up outcomes were compared between the two groups.

**Results:**

Fifty-three adolescents, including 21 with CVT, were included (mean age: 15.2 ± 1.8 years; females, 54.7%). The CVT group was more likely to experience seizures (*P* = 0.028) and deterioration (28.6% vs. 6.2%, *P* = 0.047) during hospitalization than the non-CVT group. Poor short-term outcomes, based on the modified Rankin Scale (mRS) score at discharge, were more common in adolescents with CVT (*P* = 0.007). The proportions of patients showing edema (42.9% vs. 6.2%, *P* = 0.004) and mass effect (*P* = 0.015) were significantly higher in the CVT group. Recanalization was observed in 61.9% and 82.1% of the patients in the CVT and non-CVT groups, respectively, during the first imaging review (median, 22 days). After a 12-month follow-up, female adolescents had more frequent resident secondary headaches than male adolescents (52.9% vs. 12.5%; *P* = 0.014).

**Conclusions:**

Cortical vein involvement in adolescents with CVST was associated with a higher risk of epilepsy at presentation, deterioration during hospitalization, edema, and mass effect on acute imaging. Moreover, cortical vein involvement may lead to worse short-term outcomes. Sex differences require consideration in etiological analyses and prolonged follow-ups.

**Supplementary Information:**

The online version contains supplementary material available at 10.1186/s12959-023-00521-3.

## Introduction

Cerebral venous sinus thrombosis (CVST) is a rare cerebrovascular disease that can affect patients of any age, even from the neonatal period to adolescence [1]. Data from previous studies suggest that the incidence of CVST in neonates was higher (2.6 per 100,000 neonates per year) than that in adults (1.3–1.5 per 100,000 adults per year in high-income countries) [2–4]. It was also reported that neonates are more likely to have a poor prognosis than older children [5, 6]. However, a recent study highlighted that adverse outcomes 1 year after CVST are more frequent in adolescents than in adults [7], suggesting that juvenile CVST is complex with a specific epidemiology, even though sex is thought to play a role. As a critical period in the transition from infancy to adulthood, adolescence is a stage in which neurological disorders must be considered separately. Nevertheless, few studies have been devoted to CVST during adolescence, not to mention the association of adolescence with cortical vein thrombosis (CVT), one of the most neglected clinical diagnoses. Although studies in adults and children have highlighted the involvement of CVT in venous infarction and parenchymal hemorrhage [8–10], the significance of CVT in adolescents remains unknown.

Therefore, the objective of this study was to determine whether adolescent CVST patients with and without CVT showed differences in clinical and paraclinical features, associated conditions, risk factors, and imaging characteristics, to indirectly assess the differences that affect the prognosis of adolescent CVST and provide useful data for clinicians to manage these patients.

## Methods

### Patient identification and selection

In this retrospective cohort study, patients with CVST were identified from the stroke registry of Xuanwu Hospital, Capital Medical University. Adolescent patients (age, ≥ 10 to ≤ 18 years) who experienced their first episode of CVST between January 2015 and August 2022 were consecutively included in this study. The diagnosis of CVT was based on various imaging modalities, including magnetic resonance imaging (MRI), contrast-enhanced (CE) magnetic resonance venography (MRV), computed tomography (CT), computed tomography venography (CTV), and digital subtraction angiography (DSA). All diagnoses were made jointly by the clinicians (JGD and RM) and radiologist (KYZ), who had experience of over ten years in diagnosing CVST. The cutoff ages for adolescence were chosen according to the World Health Organization and Chinese developmental psychology definitions. This study was approved by the Ethics Committee of Xuanwu Hospital, Capital Medical University, China.

Patients of both sexes were included, while patients with isolated CVT were excluded because their inclusion may have increased the bias caused by the use of CVT as a grouping variable.

### Data collection

Baseline data were reviewed and collected for all patients, including information regarding demographic characteristics, clinical presentation (headache, intracranial hypertension, papilledema, seizures, visual disturbances, motor deficits, aphasia, and mental status disorders [cognitive disturbances, including abnormal alertness and orientation]), initial medication, and major treatment. CVST-associated conditions such as malignant hemopathy/cancer, myeloproliferative neoplasms, paroxysmal nocturnal hemoglobinuria (PNH), systemic diseases (Behçet disease, antiphospholipid syndrome, systemic lupus erythematosus [SLE], and hyperthyroidism), thrombophilia (protein C, S, or antithrombin III deficiency, hyperfibrinogenemia, and homocysteinemia), the general causes of hypercoagulability (anemia, nephrotic syndrome, and pregnancy/puerperium), infection (mastoiditis, sinusitis, central nervous system infections, and other infections), and noninfectious local causes (head trauma/surgery and dural fistula) were included. The risk factors for CVST (obesity and the use of combined oral contraception) were also recorded.

Parenchymal lesions were assessed using a combination of MRI and CT techniques, including unenhanced CT, T2-weighted imaging (T2-WI), fluid-attenuated inversion recovery (FLAIR), diffusion-weighted imaging (DWI), and apparent diffusion coefficient (ADC) sequences [11, 12]. The identified parenchymal changes were then classified into four distinct subgroups: edema, venous infarction, hemorrhage, and mass effect. (1) Edema can be detected on CT or MR images by observing focal irregularities in attenuation or signal intensity. These abnormalities can be further categorized into two types: vasogenic edema, characterized by elevated ADC values that are presumed to be associated with venous congestion, and cytotoxic edema, characterized by reduced ADC values attributed to cellular energy disruption, as revealed by DWI techniques [13]. (2) Venous infarction is defined as the presence of brain parenchymal lesions that encompass local edema as well as petechial or confluent cerebral hemorrhage [14]. (3) Hemorrhagic lesions typically exhibit hyperattenuation on CT, hyperintensity on T2-WI and FLAIR sequences while appearing hypointense on T1-weighted imaging (T1-WI). These lesions often display a cortico-subcortical topography, do not follow an arterial distribution, and frequently exhibit a distinct hypointense component on T2-WI imaging. Such components are often observed as flaky, multinodular subcortical zones [15]. (4) Mass effect refers to the localized brain swelling or bleeding that manifests as various observable characteristics, including sulcal effacement, reduced visibility of cisterns, compression, deformation of one or both lateral ventricles, displacement of midline structures, and potential cerebral herniation, which could be recognized on unenhanced CT [16].

The type of onset was categorized according to the interval from symptom onset to admission as acute (< 48 h), subacute (between 48 h and 30 days), or chronic (> 30 days). The time before diagnosis (defined as the delay between symptom onset and diagnosis confirmed by imaging) was recorded when available.

The Modified Rankin Scale (mRS) scores at admission and discharge, date of initial review head CTV/MRV/MRI/magnetic resonance black blood thrombus imaging (MRBTI) and the degree of recanalization recorded on this date, and deterioration during hospitalization were recorded. Deterioration was defined as the occurrence of any new or worsening neurological symptoms observed from the day of CVST diagnosis to the day of discharge. These symptoms encompassed the aggravation of headache accompanied by thrombotic progression or the identification of new brain lesions confirmed through imaging reexamination. Additionally, deterioration included new-onset or progressive visual disturbances, new-onset or progressive seizures, a decrease in Glasgow Coma Scale scores, and the emergence of new symptoms related to focal neurological deficits, such as sensory changes, mental status alterations, aphasia, or a decline in motor function.

### Subgroup comparisons

Within our cohort, we compared some characteristics according to sex or the presence of CVT.

### Follow-up and clinical outcomes

Regular follow-up assessments were conducted for 12 months after discharge. Follow-up and outcome data were collected using a standardized questionnaire during clinical outpatient visits. Sinus recanalization status was assessed by an experienced neuroradiologist who was blinded to the clinical follow-up data and classified as no, partial, or complete recanalization in accordance with the proposed criteria (excluding patients with isolated CVT or incomplete imaging data) (11). The mRS score at the follow-up assessment performed 12 months after discharge was used as the primary endpoint of efficacy, with an mRS score > 1 indicating that the patient had not completely recovered and an mRS score ≤ 1 indicating a favorable outcome. CVST recurrence and resident symptoms were also recorded.

### Statistics

Continuous variables were expressed as mean ± standard deviation (SD) or median with interquartile range (IQR), and categorical variables were expressed as percentages. The values of the measured parameters were checked for conformity to a normal distribution by using the Kolmogorov–Smirnov test before statistical analysis. Bivariate analysis with the t-test or Mann–Whitney U test for continuous variables and the chi-square test for categorical variables were used to identify potential variables associated with CVT or sex. All statistical analyses were performed using R Statistical Software version 4.2.0 (https://www.r-project.org/, The R Foundation) and Free Software Foundation statistics software, version 1.7.1. Statistical significance was set at P < 0.05.

## Results

### Demographic data

Between 2015 and 2022, 57 patients aged ≥ 10 to ≤ 18 years with a verified diagnosis of CVST were identified. Of these, four patients were excluded because they showed isolated CVT. Baseline data are shown in Table 1. Fifty-three patients with CVST were eligible for this study. The mean age at CVT occurrence was 15.2 ± 1.8 years. A small majority of the patients (54.7%) were male.

**Table 1:** Demographic data and clinical characteristics of CVT in Adolescents

	Total (n = 53)	Non-CVT (n = 32)	CVT (n = 21)	*P* value
Gender(Male), n(%)	29 (54.7)	20 (62.5)	9 (42.9)	0.16
Mean age(y), Mean ± SD	15.2 ± 1.8	15.4 ± 1.6	15.0 ± 2.0	0.403
BMI, Mean ± SD	22.6 ± 5.2	22.1 ± 5.2	23.4 ± 5.2	0.376
Type of onset*, n(%)	47 (88.7)	28 (87.5)	19 (90.5)	1
Mean delay between first symptom and diagnosis(SD), d	7.0 ± 14.9	7.7 ± 16.6	5.8 ± 12.1	0.668
mRS 0–1 at admission, n(%)	42(79.2)	29(90.6)	13(61.9)	0.017
Deterioration during hospitalization, n(%)	8 (15.1)	2 (6.2)	6 (28.6)	0.047
mRS 0–1 at discharge, n(%)	48 (90.6)	32 (100)	16 (76.2)	0.007
**CVST-associated conditions, n(%)**	47 (88.7)	27 (84.4)	20 (95.2)	0.384
Malignant hemopathy/cancer	1 ( 1.9)	0 (0)	1 (4.8)	0.396
Myeloproliferative neoplasms	2 ( 3.8)	1 (3.1)	1 (4.8)	1
PNH	2 ( 3.8)	0 (0)	2 (9.5)	0.152
Systemic disease	12 (22.6)	8 (25)	4 (19)	0.743
Thrombophilia	24 (45.3)	14 (43.8)	10 (47.6)	0.782
General cause of hypercoagulability	16(30.2)	10(31.2)	6(28.6)	0.835
Infection	16 (30.2)	10 (31.2)	6 (28.6)	0.835
Noninfectious local cause	4 ( 7.5)	2 (6.2)	2 (9.5)	1
**CVST risk factors, n(%)**	14 (27.5)	5 (16.7)	9 (42.9)	0.039
Oral contraception	1 ( 1.9)	0 (0)	1 (4.8)	0.396
Obesity	13 (25.5)	5 (16.7)	8 (38.1)	0.084
**Clinical presentation, n(%)**				
Headache, ICP ↑†	35 (67.3)	19 (61.3)	16 (76.2)	0.261
Papilloedema	33 (62.3)	20 (62.5)	13 (61.9)	0.965
Visual disturbance	9 (17.0)	5 (15.6)	4 (19)	1
Seizure	14 (26.4)	5 (15.6)	9 (42.9)	0.028
Focal neurological deficits‡	12 (22.6)	7 (21.9)	5 (23.8)	1
Encephalopathy§	11 (20.8)	7 (21.9)	4 (19)	1

Abbreviations: CVT, cortical vein thrombosis; SD, standard deviation; BMI, body mass index; mRS, modified Rankin Scale; CVST, cerebral venous thrombosis; PNH, paroxysmal nocturnal hemoglobinuria; ICP, intracranial pressure

* acute/subacute

† Elevated intracranial pressure with opening pressure of > 25 cm H_2_O

‡ Motor deficits, aphasia, sensory deficits, hemianopia

§ Neuropsychological symptom, disturbance of consciousness, coma

### Clinical characteristics

Twenty-one (39.6%) patients had CVT (CVT group), while 32 (60.4%) patients had CVST without CVT (non-CVT group). Table 1 compares the clinical characteristics of the two patient groups. The pattern of symptom onset showed no difference between male and female adolescents or between the CVT and non-CVT groups; the onset was mostly acute/subacute and rarely chronic. The mean delay between the onset of the first symptom and diagnosis was > 7 days (Table 1).

Headache with elevated intracranial pressure was the leading symptom in all patients, followed by papilledema (29.5%) and seizures (26.4%). Patients with CVT were more likely to present with epilepsy (*P* = 0.028). During hospitalization, patients in the CVT group had a higher risk of deterioration than patients in the non-CVT group (28.6% vs. 6.2%, *P* = 0.047), with 66.7% showing aggravation of headache and new thrombi and 33.3% showing increased epilepsy. Comparatively, the CVT group exhibited higher mRS scores both at admission (P = 0.017) and at discharge (P = 0.007) in comparison to the non-CVT group. Notably, there were no other observed differences related to sex in terms of clinical symptoms, apart from the differences in mRS scores at admission (Supplementary Table 2).

Forty-seven patients (88.7%) had at least one definite CVST-associated condition, and 21 patients had at least one CVST risk factor, which was not significantly different between the CVT group and non-CVT group. However, it is worth noting that the CVT group had more CVST risk factors, which was statistically significant (P = 0.037, Table 1). Female adolescents showed a significantly higher prevalence of anemia than male adolescents (13 [54.2%] vs. 1 [3.4%], *P* < 0.001), whereas homocysteinaemia was more frequent in male patients than in female patients (*P* = 0.038, Supplementary Table 2).

### Imaging characteristics

**Fig. 1:**
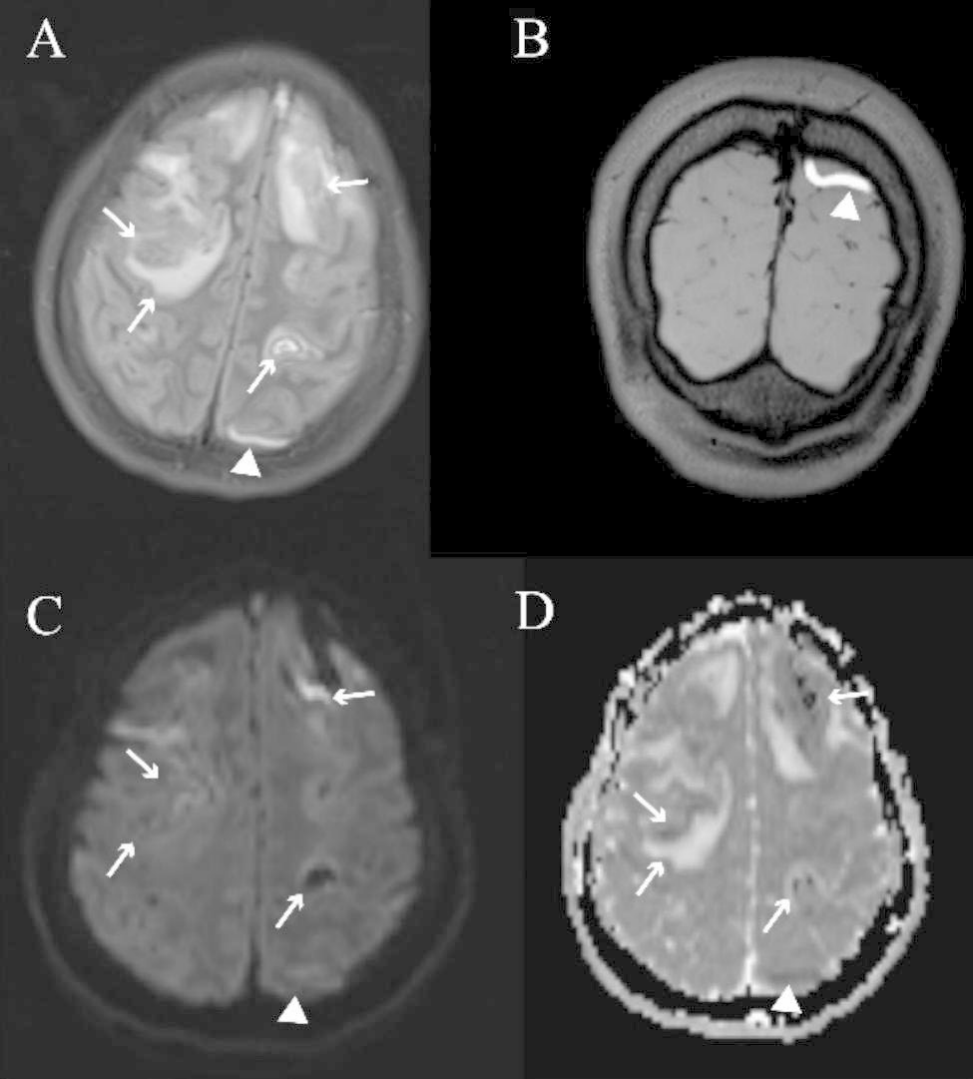
Typical images of cortical vein thrombosis on conventional magnetic resonance imaging and 3D-T1-SPACE scans in a 17-year-old female patient with cerebral venous sinus thrombosis. The fluid-attenuated inversion recovery (FLAIR, **A**) image shows a cord-like hyperintensity (white arrowheads, representing a subacute thrombosis) of the left cortical vein, which presents as restricted diffusion (white arrowheads) on the diffusion-weighted image (DWI, **C**) and apparent diffusion coefficient map (ADC, **D**), consistent with the thrombosed cortical veins on 3D-T1-SPACE (white arrowheads, **B**). Venous infarction and parenchymal hemorrhage can be observed as mixed signals on FLAIR, DWI, and ADC scans (white arrow)

Patients, regardless of the presence or absence of CVT, exhibited a comparable distribution of thrombi across multiple locations (Table 2); the most common sites of thrombosis in all patients were the superior sagittal sinus (67.9%) and right transverse sinus (60.4%). Most thrombosis locations were similar between male and female patients and between CVT and non-CVT adolescents. Specifically, the prevalence of straight sinus thrombosis was significantly higher in the non-CVT group compared to the CVT group (*P* = 0.04). Furthermore, right transverse sinus and right internal jugular vein thrombosis demonstrated a higher incidence in the male group in comparison to the female group (*P*_1_ = 0.049, *P*_2_ = 0.02) (Supplementary Table 3). In comparison with the non-CVT group, the CVT group was more likely to show edema (*P* = 0.004) and mass effect (*P* = 0.015) and had a slightly higher proportion of cases showing venous infarction. Four patients who showed mass effects had thrombosed cortical veins, of whom two female patients had a severe mass effect in which the encephalocoele was compressed and the basal midline of the brain was displaced (Fig. 1). Recanalization, including complete or partial resolution of thrombosis, occurred in 61.9% of the patients in the CVT group and 82.1% of those in the non-CVT group during the first imaging review, which was performed at a median interval of 22 days after onset (IQR, 13–31), although there was no statistically significant difference.

**Table 2:** Imaging Characteristics of CVT in Adolescents

	Total (n = 53)	Non-CVT (n = 32)	CVT (n = 21)	*P* value
**Imaging characteristics at admission, n(%)**				
Edema	11 (20.8)	2 (6.2)	9 (42.9)	0.004
Venous infarction	15 (28.3)	6 (18.8)	9 (42.9)	0.057
Parenchymal hemorrhage	14 (26.4)	6 (18.8)	8 (38.1)	0.118
**Mass effect, n(%)**				0.015
Mild	2 ( 3.8)	0 (0)	2 (9.5)	
Severe	2 ( 3.8)	0 (0)	2 (9.5)	
**Acute sites of thrombosis, n(%)**				
Superior sagittal sinus	36 (67.9)	19 (59.4)	17 (81)	0.1
Right transverse sinus	32 (60.4)	22 (68.8)	10 (47.6)	0.124
Left transverse sinus	24 (45.3)	16 (50)	8 (38.1)	0.394
Right sigmoid sinus	23 (43.4)	15 (46.9)	8 (38.1)	0.528
Left sigmoid sinus	14 (26.4)	9 (28.1)	5 (23.8)	0.727
Right internal jugular vein	15 (28.3)	9 (28.1)	6 (28.6)	0.972
Left internal jugular vein	10 (18.9)	7 (21.9)	3 (14.3)	0.722
Straight sinus	13 (24.5)	11 (34.4)	2 (9.5)	0.04
Torcular herophili	15 (28.3)	8 (25)	7 (33.3)	0.51
Multiple lesions*, n(%)	24 (45.3)	17 (53.1)	7 (33.3)	0.157
Initial imaging review days, (IQR)	22.0 (13.0, 31.0)	24.0 (13.8, 58.5)	21.0 (12.0, 23.0)	0.097
Initial recanalization, n(%)	36/49(73.5)	23/28(82.1)	13/21(61.9)	0.112

Abbreviations: CVT, cortical vein thrombosis; IQR, interquartile range

* Number of sites involved more than 3

### Treatment and prognosis

Most patients in the cohort received anticoagulation therapy during the acute/subacute period (Supplementary Table 1), with a slightly higher proportion observed in male adolescents (Supplementary Table 4). They were initially treated with low-molecular-weight heparin at adjusted doses for 10–14 days immediately after diagnosis, followed by oral anticoagulants for 3–6 months or longer according to the standard treatment protocol in the current guidelines [11]. Warfarin and novel oral anticoagulants (NOACs) were commonly used as long-term anticoagulants in 69.2% and 23.1% of the patients, respectively. The median duration of anticoagulation therapy in our cohort was 10.5 months, with no difference between the CVT and non-CVT groups.

After a 12-month follow-up (IQR, 10–15 months), 91.9% of the patients had a favorable outcome with an mRS score of 0–1. Furthermore, the long-term recanalization rates were relatively consistent among patients, irrespective of whether they had CVT or not (Supplementary Table 1). A higher proportion of female adolescents than male adolescents had a resident secondary headache (52.9% vs. 12.5%; *P* = 0.014, Supplementary Table 2). Recurrent venous thrombosis was more frequent in male patients (n = 5) than in female patients (n = 2). Among them, three male patients and one female patient had homocysteinemia, of whom the female patient was also diagnosed with PNH and anemia. The other female patient also had anemia. One male patient had antiphospholipid syndrome, while the other male patient was diagnosed with arteriovenous malformation, which resulted in delayed brain hemorrhage.

## Discussion

To the best of our knowledge, this is the first cohort study of adolescents with CVST to investigate the clinical manifestations and imaging outcomes of CVT. Despite the variable incidence of cortical vein involvement in pediatric (17–24%) and adult (61.4%) patients [8, 10], the calculated CVT incidence was 42.9% in our study, which can be explained as an age effect since adolescence represents a continuous transition between childhood and adulthood.

In our adolescent cohort, it was observed that the majority of patients, irrespective of whether they had cortical vein-involved thrombosis, experienced headaches of varying degrees accompanied by elevated intracranial pressure (ICP). However, adolescent patients with CVT exhibited higher mRS scores upon admission and faced an increased risk of deterioration during their hospital stay. This higher risk of deterioration directly contributed to worse short-term outcomes at the time of discharge. Furthermore, these findings were consistent with a lower recanalization rate observed during the initial image review after discharge. This suggests that clinicians should give particular attention to the presence of concomitant cortical vein involvement in adolescent patients with CVST, as it may directly impact the initial disease severity, the progression of symptoms during the hospital stay, and overall prognosis. One typical manifestation of adolescent CVT was seizure (42.9%); adolescents with CVT were more likely to present with epilepsy than those without CVT (*P* = 0.028), and the incidence of seizures was similar to that in adults but less than that in young children (44.4% vs. 58%) [8, 10].

Restricted diffusion was a common finding in a recent study on pediatric patients with CVST [8]. Moreover, as demonstrated in adults and children, patients with cortical vein involvement are more likely to develop venous infarction, parenchymal hemorrhage, and poor outcomes [8, 10]. CVT was strongly correlated with edema and mass effect in our study, and the cortical veins were involved in all four patients presenting with mass effect. The proportions of venous infarction and parenchymal hemorrhage in the CVT group were higher than those in the non-CVT group, although no significant differences were observed, as reported in previous studies. In addition to the age at thrombosis, which may be a contributory factor, isolated CVT can cause a significantly higher proportion of parenchymal brain lesions (81%), which has been demonstrated in a systematic review in which most participants were adults [17]. However, in contrast to previous studies, our study excluded this type, which may have a specific pathological mechanism that may be attributed to the slightly different results.

Most patients in our CVST cohort received initial anticoagulation treatment and achieved eventual recanalization at the 12-month follow-up, despite the mean delay of > 1 week between the first symptom and diagnosis. Interestingly, the risks of recurrence and resident neurological dysfunction were evenly distributed between the CVT and non-CVT groups. Seven children with recurrence had CVST-associated diseases, highlighting the need for systematic and extensive etiological examinations in adolescent patients. Ritchey et al. reported that thrombosis of the cortical veins may be associated with residual headache, seizures, and neurological disability between 6 weeks and 6 months in pediatric CVST [8]. Besides, in our cohort, no cases of residual epilepsy were observed during the 12-month follow-up, indicating that the impact of CVT may be more prominent in the acute phase of CVST rather than in long-term prognosis. These findings highlight the importance of implementing long-term follow-up programs for adolescent patients with CVT to address potential concerns beyond the acute phase.

Since our study population consisted of adolescents, it also showed the effects of sex; male and female patients showed differences in CVST-associated conditions, which were probably related to puberty and hormonal changes. In assessments of long-term outcomes, male adolescents tended to experience more recurrences. Secondary headache was observed in over half of female adolescents, similar to the results reported in another recent adolescent study [7]. In addition, some patients reported memory impairment and an inability to continue learning, suggesting that cognitive and other neuropsychological changes may be involved in CVST sequelae in future follow-ups of adolescents.

The major limitations of our study include its small sample size, retrospective nature, and lack of comprehensive follow-up data. In addition, all patients in this study were selected from the registry of hospitalized patients at our center, which may have increased the selection bias. Furthermore, the influence of isolated CVT in adolescents was not investigated, which warrants future prospective studies with larger sample sizes.

## Conclusions

Our study suggests that despite showing excellent overall survival rates, adolescents with cortical vein involvement have a higher risk of epilepsy at presentation, deterioration during hospitalization, edema and mass effect on acute imaging, and may further result in worse short-term outcomes. The findings also indicate the need to account for sex differences in etiological analyses and prolonged follow-ups.

## Electronic supplementary material

Below is the link to the electronic supplementary material.

Supplementary Material 1

Supplementary Material 2

Supplementary Material 3

Supplementary Material 4

## Data Availability

Data available on request from the authors.
